# Mycorrhizal inoculation effects on growth and the mycobiome of poplar on two phytomanaged sites after 7-year-short rotation coppicing

**DOI:** 10.3389/fpls.2022.993301

**Published:** 2022-10-28

**Authors:** Lisa Ciadamidaro, Stéphane Pfendler, Olivier Girardclos, Cyril Zappelini, Philippe Binet, Valerie Bert, Damase Khasa, Damien Blaudez, Michel Chalot

**Affiliations:** ^1^ Chrono-environnement UMR6249, CNRS, Université Bourgogne Franche-Comté, Besançon, France; ^2^ Laboratoire Chrono-environnement UMR6249, CNRS, Université Bourgogne Franche-Comté, Besançon, France; ^3^ Agroécologie, INRAE, Institut Agro, Univ. Bourgogne, Univ. Bourgogne Franche-Comté, Dijon, France; ^4^ INERIS, Clean Technologies and Circular Economy Unit, SIT, Parc Technologique Alata, BP2, Verneuil-en- Halatte, France; ^5^ Centre for Forest Research and Institute for Systems and Integrative Biology, Université Laval, Québec, QC, Canada; ^6^ Universiteí de Lorraine, CNRS, LIEC, Nancy, France; ^7^ Université de Lorraine, Faculté des Sciences et Technologies, Nancy, France

**Keywords:** ectomycorrhizal, endophytic fungi, Populus, phytomanagement, Skado clone, fungal diversity, trace elements

## Abstract

**Aims:**

Afforestation of trace-element contaminated soils, notably with fast growing trees, has been demonstrated to be an attractive option for bioremediation due to the lower costs and dispersion of contaminants than conventional cleanup methods. Mycorrhizal fungi form symbiotic associations with plants, contributing to their tolerance towards toxic elements and actively participating to the biorestoration processes. The aim of this study was to deepen our understanding on the effects of mycorrhizal inoculation on plant development and fungal community at two trace-element contaminated sites (Pierrelaye and Fresnes-sur-Escaut, France) planted with poplar (*Populus trichocarpa* x *Populus maximowiczii*).

**Methods:**

The 2 sites were divided into 4 replicated field blocks with a final plant density of 2200 tree h^-1^. Half of the trees were inoculated with a commercial inoculum made of a mix of mycorrhizal species. The sites presented different physico-chemical characteristics (e.g., texture: sandy soil *versus* silty-loam soil and organic matter: 5.7% *versus* 3.4% for Pierrelaye and Fresnes-sur-Escaut, respectively) and various trace element contamination levels.

**Results:**

After 7 years of plantation, inoculation showed a significant positive effect on poplar biomass production at the two sites. Fungal composition study demonstrated a predominance of the phylum Ascomycota at both sites, with a dominance of *Geopora Arenicola* and *Mortierella elongata*, and a higher proportion of ectomycorrhizal and endophytic fungi (with the highest values observed in Fresnes-sur-Escaut: 45% and 28% for ECM and endophytic fungi, respectively), well known for their capacity to have positive effects on plant development in stressful conditions. Furthermore, Pierrelaye site showed higher frequency (%) of mycorrhizal tips for ectomycorrhizal fungi (ECM) and higher intensity (%) of mycorrhizal root cortex colonization for arbuscular mycorrhizal fungi (AMF) than Fresnes-sur-Escaut site, which translates in a higher level of diversity.

**Conclusions:**

Finally, this study demonstrated that this biofertilization approach could be recommended as an appropriate phytomanagement strategy, due to its capacity to significantly improve poplar productivity without any perturbations in soil mycobiomes.

## Introduction

Phytomanagement is now widely applied worldwide for the remediation of soils that are contaminated by trace elements (TE) within the context of phytostabilization (Šimek et al., 2017; Burges et al., 2018) and phytoextraction (Sessitsch et al., 2013) scenarios. Rhizosphere microorganisms can actively participate in these phytoremediation processes and could be useful in extending the application of phytobial remediation (Berendsen et al., 2012; Thijs et al., 2016; Dąbrowska et al., 2017; Touceda-Gonzaílez et al., 2017; Xue et al., 2018; Bonito et al., 2019; Yan et al., 2020). Microorganisms are ubiquitous across environments and play key roles within ecosystems and in mutualistic associations with host organisms. Plant-associated microorganisms are known to assist their hosts with key functions, such as water and nutrient absorption, driving material cycles, and alleviating biotic and abiotic stresses (Morte et al., 2001; Hill et al., 2010; Li et al., 2013). The biodiversity of soil microbial communities is increasingly recognized as a major factor contributing to human health, both directly by limiting the spread of potential pathogens (Berendsen et al., 2012), and indirectly by contributing to processes that provide clean air and water, and healthy food (Wall et al., 2015). Fungi are one of the most abundant components of the soil microflora; the diversity of fungal communities is influenced, in turn, by a variety of climatic and edaphic factors (Tedersoo et al., 2014; Maestre et al., 2015). Mycorrhizal fungi are known to form symbiotic associations and improve plant health (Van der Heijden et al., 2015), but the lack of host specificity between different species for arbuscular mycorrhizal fungi, or even different individuals within a species, may induce different responses and ecological effects (Munkvold et al., 2004; Berruti et al., 2016). Despite having key functions in terrestrial ecosystems, information on the dominant soil fungi and their ecological preferences at the global scale is lacking. A study of Egidi et al. (2019) demonstrated that generalist Ascomycota, dominate soils globally and that generalist fungi point at a significantly higher number of genes related to stress-tolerance and resource uptake in the dominant fungi, suggesting that they might be better in colonising a wide range of environments. Due to their potential functional complementarity, high mycorrhizal diversity is expected to be more beneficial to host plants than is low diversity (Johnson et al., 2003; Maherali and Klironomos, 2007).

Over many decades mining and smelting operations across Europe have resulted in large numbers of sites that have been contaminated by TE (Quercia et al., 2006; Roccotiello et al., 2015). Soil metal concentrations on these sites frequently reach levels that are toxic both to plants (Cobbett, 2003) and to microorganisms (Giller et al., 1998; Dai et al., 2004), which can especially reduce the diversity of the latter (Moffett et al., 2003). Differences in metal tolerance among organisms also can lead to changes in species composition and genetic diversity (Smit et al., 1997; Turpeinen et al., 2004; Colpaert et al., 2011; Yamaji et al., 2016; Torres-Cruz et al., 2018). For example, severe zinc (Zn) pollution can trigger differential selection towards increased Zn tolerance in ECM fungi, as has been shown by Colpaert et al. (2004; 2005) and Adriaensen et al. (2006) in *Suillus* isolates. Therefore, mycorrhizal mediation of plant performance can be particularly important in very stressful environments (Jan and Parray, 2016), such as soils that have been contaminated with TE. In this regard, a proposed strategy for the remediation of stressed and degraded environments is based upon phytotechnologies involving biological processes such as plant-microbe interactions (Glick, 2010; Ali et al., 2013; Kaur and Garg, 2018), through the inoculation of mycorrhizal strains to improve the quantity and diversity of the fungal community.

*Populus* species have been successfully used for the revegetation of lands heavily contaminated with TE due to their high tolerance to excessive TE concentrations (Hur et al., 2011; Guerra et al., 2011; Castro-Rodríguez et al., 2016; Chandra et al., 2016; Barra Caracciolo et al., 2020). These species are also characterized by fast growth rates and their ability to stabilize the soil surface, reduce wind and water erosion of the soil, and decrease the risk of contaminant leaching (Domiínguez et al., 2009; Ciadamidaro et al., 2014). In addition, understanding the interactions between poplar and fungi can be of particular socio-economic importance, as poplars are currently cultivated for pulp and paper production (Tuskan, 1998; Dinus et al., 2001) and have the potential as a feedstock for cellulose-derived biofuels (Tuskan et al., 2006; Sannigrahi et al., 2010; Castro-Rodríguez et al., 2016). For these reasons, numerous efforts have been made to characterize the poplar microbiome using high throughput sequencing (Gottel et al., 2011; Danielsen et al., 2012; Shakya et al., 2013; Cregger et al., 2018).

Our previous papers have emphasized genotypic variation in poplar growth at a moderately contaminated site (PHYTOPOP trial). We also examined element accumulation, and hence, nutrient export capacities of poplar (Pottier et al., 2015; Chalot et al., 2020). In this experiment, the Skado (*P. trichocarpa* Torrey & A. Gray ex. Hook x *P. maximowiczii* A. Henry) genotype maintained a similar yield (after 7 years growing) of about 7 oven-dry ton (odt) ha^-1^ year^-1^ and was among the most productive poplar genotypes at this site (Chalot et al., 2020). This genotype was chosen in 2011 for a new phytomanagement plantation at the same site (Pierrelaye) and at a new site (Fresnes-sur-Escaut) within the BIOFILTREE project (Ciadamidaro et al., 2017). Inoculation with mycorrhizal fungi significantly increased the biomass production of the Skado clone at the two sites after 2 and 4 years of cultivation, respectively (2013 and 2015), despite striking differences in soil structure and contamination. However, the effect of inoculation on the soil fungal communities was not investigated in these previous papers (Ciadamidaro et al., 2017; Phanthavongsa et al., 2017).

On this regard, for this study, we hypothesized that inoculation of poplars with a mycorrhizal consortium (including ECM and AMF) would shape the fungal communities. We combined high-throughput sequencing technologies with plant and soil analyses to understand the relationships among plants, soil properties, and microbial characteristics along restoration strategies. We also determined the composition and diversity of fungal communities at two contrasting phytomanaged sites, to better understand their ecological roles in TE-exposed ecosystems. Lastly, we completed our previous dendrometric measurements after 7 years (Ciadamidaro et al., 2017), to determine whether inoculum introduced during planting had a long-term effect on the growth of poplar.

## Materials and methods

### Site description and experimental design

The location and history of the two demonstration sites have been described in detail in a previous paper (Ciadamidaro et al., 2017). Briefly, one site is located in Pierrelaye (Ile-de-France, France, 49°1’47’’ N, 2°10’29’’ E), while the other is located at Fresnes-sur-Escaut (Hauts-de-France, France, 50°25’47” N, 3°35’7” E). Continuous irrigation of the Pierrelaye (P) site with raw wastewaters for more than a century has led to polymetallic pollution, which is characterized by Pb, Cu, Zn and Cd concentrations that are 10-fold higher than in a non-irrigated reference soil (Lamy et al., 2006). The sediment deposit site of Fresnes-sur-Escaut (F) is linked to past mining and metal-smelting activities, leading to contamination with TE, such as Cd, Zn, As and Pb.

The properties of the two study soils represent two contrasting environments. The Pierrelaye site has a sandy soil (> 80% sand), whereas the sediment of the Fresnes-sur-Escaut site has a silt-loam texture with higher mean organic matter (OM) content than the former (5.7% vs 3.4%; Phanthavongsa et al., 2017). However, bulk pH_H2O_ of the two soils was similar and slightly alkaline, i.e., pH 7.7 and 7.6 for Pierrelaye and Fresnes, respectively.

Four replicate field plots were established in February and March 2011 for Pierrelaye and Fresnes-sur-Escaut, respectively, using cuttings of poplar clone Skado (*P. trichocarpa* Torrey & A. Gray ex. Hook x *P. maximowiczii* A. Henry; both parents are section *Tacahamaca*). The unrooted poplar cuttings were about 1.5 m length, and were planted at an initial spacing of 1.8 m x 2.75 m for a final density of 2200 stems ha^-1^. The criteria for selecting the Skado clone were determined in a previous assay (Phytopop; see Pottier et al., 2015) and based on the following traits: i) high growth yield and ii) low TE accumulation capacities. Half of the whips were inoculated (4 inoculated plots per clone) with a mycorrhizal consortium (as described in next section), while the other half was left uninoculated (4 control plots per clone).

### Inoculation protocol

The fungal inoculum consisted of the AM fungus *Rhizophagus irregularis* DAOM 197198 strain (Agronutrition, Toulouse, France) and two commercial preparations, i.e., Symbivit^®^ and Ectovit^®^, which are both produced by Symbiom (Landskroun, Czech Republic). Symbivit contains six AMF: *Rhizophagus intraradices* BEG140, *Funneliformis mosseae* BEG95, *Funneliformis geosporum* BEG199, *Claroideoglomus claroideum* BEG96, *Claroideoglomus etunicatum* BEG92, and *Glomus microaggregatum* BEG56. Ectovit contains six ECM fungi: *Hebeloma mesophaeum, Amanita rubescens, Laccaria proxima, Paxillus involutus, Pisolithus arrhizus* and *Scleroderma citrinum*. Propagule number (spores or mycelia) was 250000/L and 1800/L of Symbivit and Ectovit inocula, respectively. About 26000 propagules (25000 Symbivit + 180 Ectovit + 900 Agronutrition) were delivered to each tree, as recommended by the manufacturers.

### Soil chemical analysis

In 2011 and 2016, 10 top-soil (0-20 cm) samples were collected in each plot with a hand auger at a distance of 20 cm from the trunks, and mixed together to form a composite sample that was used for chemical analyses. From the 16 composite samples, 8 samples per site were selected based on the pseudo-total metal(oid) concentrations to perform the agronomic characterization. The samples were force air-dried at 40°C to constant mass, ground in an agate mortar (RM100, Retsch GmbH, Haan, Germany) and sieved to pass a 200 µm mesh (AS 200, Retsch GmbH). The following analyses were contracted to a private laboratory (Galys, Blois, France): particle size distribution, exchangeable Ca^+2^ (CaO), Mg^+2^ (MgO), K^+^ (K_2_O) and Na^+^ (Na_2_O); organic matter (OM) (NF ISO 14235); total nitrate (N) (NF ISO 13878); total phosphorus (P) (NF ISO 11263); total CaCO_3_ (NF ISO 10693); cation exchange capacity (CEC) (NF X 31-130); and soluble boron (B) (NF X 31-122).

Total element concentrations (Al, Cd, Fe, Mn, Pb, S, Zn) were determined using inductively coupled plasma-atomic emission spectrometry (ICP-AES, Ultima 2, Jobin Yvon Horiba), following *aqua regia* digestion according to the EN 13346 standard. To assess the analytical quality, a standard reference material (Buffalo River Sediment, SRM 2704, NIST, Gaithersburg, MD, USA) was added to the analysis following the same protocol. Recoveries were between 77 and 110%. Available element concentrations in the soil were assessed by ICP-AES after 0.01 M CaCl_2_-extraction with a sample extraction ratio of 1:10 (Houba et al., 1996). Total element concentrations have been presented by Ciadamidaro et al. (2017); available elemental concentrations in the soils are reported in **Table 1**. Microbial biomass carbon (MBC) was determined by the chloroform fumigation-extraction method (Vance et al., 1987), which is described in greater detail by Ciadamidaro et al. (2014).

**Table 1:** CaCl_2_-extractable TE concentrations of topsoil samples (0-20 cm) from the two sites (mg kg^-1^ DW), Fresnes-sur-Escaut (F) and Pierrelaye (P) and two treatments (Control, C; Inoculated, I).

Soil	(μg g^-1^ DW)						
	As	Cd	Cr	Cu	Ni	Pb	Zn
F_C	0.02 (0.00) **a**	0.01 (0.00) **a**	0.00 (0.00) **a**	0.10 (0.04) **a**	0.02 (0.00) **a**	0.01 (0.00) **a**	0.72 (0.48) **a**
F_I	0.02 (0.01) **a**	0.01 (0.00) **a**	0.00 (0.00) **a**	0.08 (0.02) **a**	0.02 (0.00) **a**	0.01 (0.02) **a**	0.63 (0.28) **a**
P_C	0.02 (0.00) **a**	0.01 (0.00) **a**	0.01 (0.00) **a**	0.16 (0.03) **a**	0.03 (0.01) **a**	0.02 (0.01) **a**	0.73 (0.37) **a**
P_I	0.02 (0.00) **a**	0.00 (0.00) **a**	0.01 (0.00) **a**	0.15 (0.04) **a**	0.02 (0.01) **a**	0.02 (0.01) **a**	0.61 (0.13) **a**

Different letters within columns indicate significant differences between the treatments (with or without inoculation) for each site (P < 0.05).

### Tree biomass measurements

Diameter at breast height (dbh, 1.3 m) and total tree height (H_tot_) were measured in late autumn of 2017 (7 growing seasons), on 384 individual trees from the 8 plots (*i.e.*, 24 trees per plot, excluding the border trees). Shoot biomass was calculated from H_tot_ and dbh measurements that were taken for each tree (Ciadamidaro et al., 2017).

### Molecular methods and sequencing

The samples were freeze-dried (RP2V, Group S.G.D., France) and ground to a homogenous powder for 3 min at 30 Hz (Mixer Mill, model MM400, Retsch GmbH, Haan, Germany). Environmental DNA from the soil samples was extracted with the PowerSoil DNA isolation Kit following manufacturer’s instructions (MoBio Laboratories, Inc., Carlsbad, CA, USA). For all samples, a purification step was performed using the Power Clean^®^ Pro DNA Clean-Up kit (MoBio Laboratories, Inc.) to enhance DNA quality. DNA quality and quantity were assessed by agarose gel electrophoresis and the Quant-iT™ PicoGreen^®^ dsDNA Assay Kit (Invitrogen, Carlsbad, CA) using an FLX-Xenius spectrofluorometer (SAFAS, Monaco). Equimolar DNA pools were produced and adjusted to 10 ng/μL. Sequencing of the fungal ITS1 region (Bonito et al., 2014) was performed on the Illumina MiSeq platform (Microsynth AG, Balgach, Switzerland). Fungus-specific primers ITS1F (CTTGGTCATTTAGAGGAAGTAA) and ITS2 (GCTGCGTTCTTCATCGATGC) (Smith and Peay, 2014) were used for PCR amplification. These primers target a short portion of the fungal ITS region, resulting in an amplicon of 300 bp, which is appropriate for Illumina sequencing.

### Data analysis

Reads were assigned to each sample according to a unique barcode. Paired reads were grouped on a contig using the *mothur* pipeline (Schloss et al., 2009). ITS1 sequences that were extracted using 18S and 5.8S motifs were searched on contigs using HMMER software (http://hmmer.org). Contigs were then filtered by length (100 < x < 350 bp), homopolymer (< 10 bp) and unknown base (< 0 bp). The contigs were pre-clustered at 100% identity.

Rare sequences that were represented by fewer than 2 reads in less than 2 different samples were removed from the analysis. Taxonomic assignments were performed using the database UNITE v7.2 (http://unite.ut.ee; Kõljalg et al., 2013). Operational taxonomic units (OTUs) were then constructed using the Needleman-Wunsch distance and average neighbour clustering (Unweighted Pair Group Mean Averaging, UPGMA) at an N-W distance of 0.03. The number of reads per sample was calculated after random sub-sampling of 30 000 reads.

### Evaluation of root colonization by fungi

Root samples were carefully washed with distilled water. About 300 randomly selected root tips per sample were examined and assessed as being mycorrhizal or non-mycorrhizal under a stereomicroscope (10 × magnification) to determine the rate of root colonization by ECM (Lacercat-Didier et al., 2016). For the evaluation of the rate of root colonization by AMF, fungal structures within the young fine roots were cleared with KOH (10%) and stained with trypan blue (5%) using the protocol of Koske and Gemma (1989). Seventy-five stained root segments were mounted on microscope slides and observed at x 100 magnification. The intensity of cortex colonization by AMF colonization (%M) was measured using a class system that had been determined by Trouvelot et al. (1986). The intensities of global root cortex colonization (%M) were calculated by using the program Mycocalc (INRA Dijon, France: https://www2.dijon.inrae.fr/mychintec/Mycocalc-prg/download.html).

### Statistical analysis

All statistical analyses, except for %M, were performed in the R environment (v. 3.6.1, R Core Team, 2019). Normality and homoscedasticity of the data were verified using Shapiro-Wilk tests and Bartlett’s tests, respectively. Comparisons of treatment means were performed using *post hoc* tests following a one-way ANOVA. Tukey’s tests were performed to separate multiple treatment means; Student’s *t*-test were applied only when there were only two subgroups. All tests were considered significant at P < 0.05.

*Pgirmess* and *Vegan* packages in R were used for the calculation of richness and diversity indices. Rarefaction curves were generated with the *rarecurve* function in *Vegan* (Oksanen et al., 2019). Two-dimensional non-metric multi-dimensional scaling (NMDS) was estimated using the Bray-Curtis method (*k = 3*) using the *metaMDS* function in the *vegan* package in R. The resulting clustering trees were paired with a heatmap of Spearman’s correlations (*r_s_*) between the relative abundances created with *heatmap.*2 from the *gplots* package. The numbers of OTUs that were shared between treatments were created using Venn diagrams implemented by *VennDiagram* in the Vegan package in R.

PCA (principal component analysis) was performed using the *FactoMinR* package (Lê et al., 2008). Data below detection-limits were considered to be null for the calculation of the principal components, and ellipses were drawn at the 95% confidence interval of the barycentre of each species.

## Results

### Soil characteristics and tree measurements

In general, concentrations of CaCl_2_-extractable TE were higher at the Pierrelaye site just for Cu concentrations (**Table 1**).

After an additional growth period of 7 years, the inoculation treatment still had a significant effect on the biomass yield of Skado, which increased by +16 and +18% at Pierrelaye and Fresnes-sur-Escaut, respectively (**Table 2**). Seven years after planting, the biomass production at the Pierrelaye site was greater than that at the Fresnes-sur-Escaut site. The fresh tree biomass increased by 101% (control) and 102% (inoculated) in Pierrelaye between 2015 and 2017, whereas it only increased by 74% (control) and 70% (inoculated) at Fresnes-sur-Escaut.

**Table 2:** Dendrometric parameters for poplars at the two experimental sites (Fresnes-sur-Escaut and Pierrelaye), inoculated or left uninoculated (control).

Site	Year	Treatment	dbh (cm)	Stat [df.] Fval. Pv	Htot (m)	Stat [df.] Fval. Pv	Fresh Biomass (kg)	Stat [df.] Fval. Pv	Yield (t/ha/year)
Fresnes-sur-Escaut	2013	Control	5.51 (1.18)		7.29 (1.49)		11.2 (5.69)		12.4
		Inoculated	6.26 (1.11)	[1/184] 28.5 ***	7.70 (1.11)	[1/184] 22.8 ***	14.8 (5.71)	[1/184] 29.1 ***	16.4
		Block		[3/184] 56.3 ***		[3/184] 67.9 ***		[3/184] 66.9 ***	
	2015	Control	9.73 (1.89)	[1/184] 7.2 **	13.2 (2.33)	[1/184] 17.1 ***	42.9 (20.9)	[1/184] 8.3 **	23.8
		Inoculated	10.6 (1.75)	[3/184] 36.8 ***	13.9 (1.87)	[3/184] 92.7 ***	52.5 (22.0)	[3/184] 44.6 ***	29.2
		Block							
	2017	Control	11.7 (2.23)		16.6 (2.65)		74.7 (35.7)		27.6
		Inoculated	12.6 (2.09)	[1/184] 2.8 .	17.4 (2.22)	[1/184] 9.6 **	89.2 (37.0)	[1/184] 2.7 ns	29.2
		Block		[3/184] 15.5 ***		[3/184] 47.3 ***		[3/184] 17.6 ***	
Pierrelaye	2013	Control	4.29 (0.96)		5.23 (0.84)		5.24 (3.66)		5.7
		Inoculated	5.03 (0.95)	[1/178] 40.3 ***	5.90 (0.71)	[1/178] 41.6 ***	8.11 (3.91)	[1/178] 44.1 ***	8.8
		Block		[3/178] 14.9 ***		[3/178] 18.6 ***		[3/178] 14.2 ***	
	2015	Control	10.2 (1.76)		11.9 (1.19)		44.3 (18.8)		24.3
		Inoculated	10.9 (1.68)	[1/178] 6.10 *	12.5 (1.20)	[1/178] 19.0 ***	52.9 (20.2)	[1/178] 9.5 **	29.4
		Block		[3/178] 1.90 ns		[3/178] 9.10 ***		[3/178] 2.3 .	
	2017	Control	12.8 (2.68)		15.7 (1.72)		74.7 (35.7)		32.8
		Inoculated	13.9 (2.57)	[1/184] 7.5 **	16.3 (1.81)	[1/184] 7.2 **	89.2 (37.0)	[1/184] 7.3 **	38.5
		Block		[3/184] 3.1 *		[3/184] 3.9 *		[3/184] 3.6 *	

Diameter at breast height (dbh), total height (H_tot_), fresh biomass and yield. Results are expressed as means ± standard error (n = 4). ANOVA values are Fval.: Fisher’s test value; df.: freedom degree; with Pv: *** (0.001), ** (0.01), * (0.05), . (0.1), ns: not significant. The values are means (± standard deviations).

**Figure 1:**
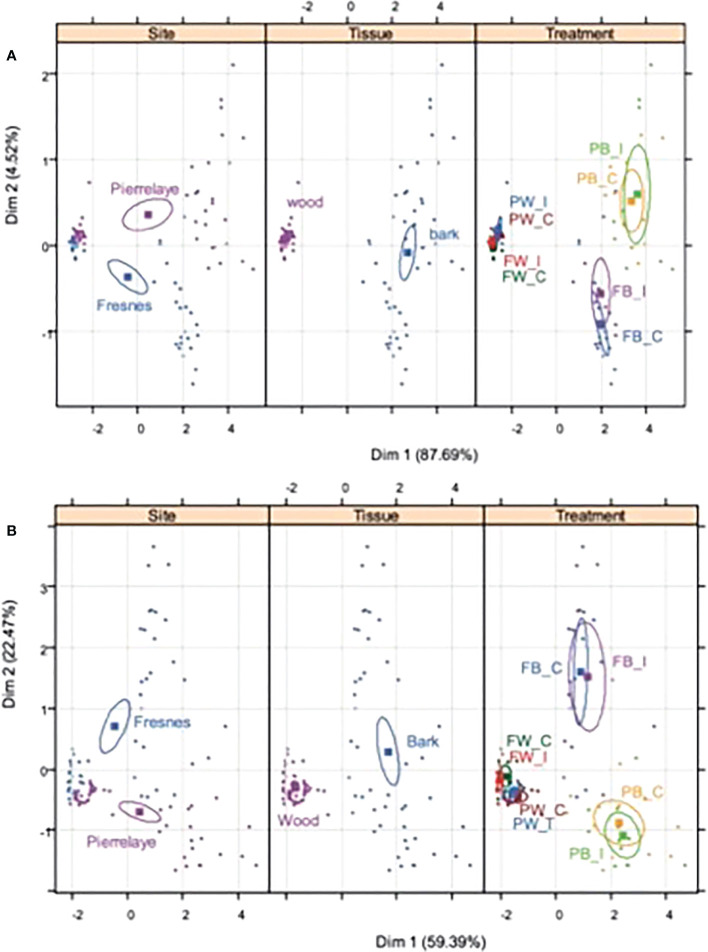
Principal component analysis showing the correlations of the nutrients **(A)** and potential toxic elements **(B)** for the two study soils (Fresnes-sur-Escaut and Pierrelaye), in two different plant tissues (wood and bark) and for the two treatments (control, C; inoculated, I). Results of the Monte Carlo permutation test demonstrate the different distributions of the nutrients and potential toxic elements (P = 0.001).

The elemental concentrations of perennial tissues of poplar were measured (**Online Resource 1**) and two PCAs were performed (**Figures 1A, B**), with nutrients and TE data, respectively. The results obtained in **Figures 1A, B** demonstrated that independent of the element category (nutrients versus TE), site and tissue variables had a strong effect on elemental concentrations, whereas the inoculation treatment did not influence element distribution on the PCA analyses. P, K, Mg, Mn, Fe and Cu concentrations were always higher in poplar tissues from Pierrelaye than from Fresnes-sur-Escaut (**Online Resource 1**). Conversely, Ca, Na, S, Cd and Zn were higher in Fresnes-sur-Escaut samples. Most elements, including Ca, P, K, Mg, Mn, S, Cd, Cu, Fe, Na and Zn, had consistently higher concentrations in the bark compared to the wood. The greatest difference was observed for Ca (16- to 18-fold difference). Yet, inoculation had no effect on element accumulation. Indeed, the ratio of these concentrations between inoculated and non-inoculated samples ranged from 0.8 to 1.1.

### Microbial biomass, richness, diversity and root colonization by fungi

**Figure 2:**
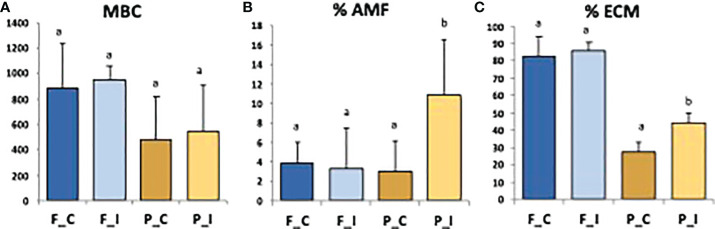
Values of microbial biomass carbon (MBC; mg C kg-1 dry soil) **(A)**, Intensity (%) of mycorrhizal root cortex colonization (% AMF) **(B)** and frequency (%) of mycorrhizal tips (% ECM) **(C)** for the two sites (Fresnes-sur-Escaut, F; Pierrelaye, P) and two treatments (control, C; inoculated, I). Different letters above the bars denote significant differences by one way ANOVA (p<0.05).

In the present study, microbial biomass carbon (MBC) did not differ significantly between samples collected at the two sites, or between treatments (**Figure 2A**). Inoculated poplars from Pierrelaye showed a significantly higher colonization rate by AMF and ECM when compared to control trees (**Figures 2B, C**). This was not the case for Fresnes-sur-Escaut. Poplars from the Fresnes-sur-Escaut site exhibited a very high percentage of ectomycorhization (> 80%); however, no statistical difference was observed between control and inoculated trees (**Figure 2C**).

Rarefaction curve analysis showed that all curves that were generated from each soil and treatment tended towards saturation, indicating that overall fungal diversity was well represented (**Online Resource 2**). Richness and diversity indices were subsequently calculated for each dataset (**Table 3**). OTU richness, Pielou evenness, and Shannon and Simpson diversity for the fungal community were generally higher for the Pierrelaye soil than for that of the Fresnes-sur-Escaut site, but these values were statistically different only in terms of the Shannon diversity index (**Table 3**). No significant change in these indices was observed between the control and inoculated soils for either site. Estimated fungal richness in OTUs was similar between the two treatments of the same soil, but higher values were observed for the Pierrelaye soil, although the differences were not significant (**Table 3**).

**Table 3:** Richness and diversity indices of the fungal communities from the two sites (Fresnes-sur-Escaut, F; Pierrelaye, P) and two treatments (control, C; inoculated, I).

	F_C	F_I	P_C	P_I
**Species richness**	458 (87.1) **a**	457 (77.0) **a**	493 (69.1) **a**	475 (62.8) **a**
**Shannon entropy**	3.79 (0.49) **a**	3.96 (0.22) **a**	4.17 (0.25) **b**	4.18 (0.17) **b**
**Shannon diversity**	48.3 (17.2) **a**	53.6 (10.4) **a**	66.3 (15.5) **b**	66.0 (10.5) **b**
**Simpson diversity**	18.3 (8.60) **a**	21.2 (5.33) **a**	27.3 (10.7) **a**	27.8 (8.24) **a**
**Pielou eveness**	0.62 (0.08) **a**	0.65 (0.04) **a**	0.67 (0.05) **a**	0.68 (0.03) **a**
**Shannon eveness**	0.11 (0.05) **a**	0.12 (0.03) **a**	0.14 (0.04) **a**	0.14 (0.03) **a**
**Simpson eveness**	0.04 (0.02) **a**	0.05 (0.02) **a**	0.06 (0.02) **a**	0.06 (0.02) **a**
**Eveness**	0.36 (0.06) **a**	0.39 (0.05) **a**	0.40 (0.09) **a**	0.41 (0.08) **a**

Data represent the mean values ( ± SE). In each row, values with the same letter do not differ significantly at P = 0.05).

### Composition and structure of fungal communities

**Figure 3:**
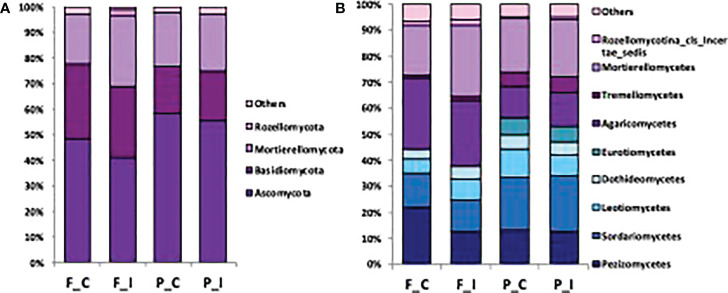
Barplots showing the fungal community composition at the phylum **(A)** and class **(B)** level in soil for each site (Fresnes-sur-Escaut, F; Pierrelaye, P) and treatment (control, C; inoculated, I).

To identify the phylogenetic diversity of the fungal communities, effective sequences have been assigned to phyla and classes. In all samples, we detected a total of 5 distinct phyla and 10 distinct fungal classes, which were unevenly distributed among the samples (**Figures 3A, B**). At both sites, the composition of the fungal community was dominated by the phylum *Ascomycota*, followed by the phyla *Basidiomycota* and *Mortierellomycota*, and was very similar between the two treatments of the same soil (**Figure 3A**). More precisely, the most abundant genders are represented by a *Geopora* sp., *Lactarius* sp., and *Mortierella* sp., respectively an Ascomycota, Basidiomycota and Mortierellomycota, that are all of them ECM. The dominance of the phylum *Ascomycota* was slightly reduced at the Fresnes-sur-Escaut site compared to the Pierrelaye site, representing respectively around 40% for Fresnes-sur-Escaut and 60% for Pierrelaye. OTUs corresponding to the phylum *Rozellomycota* were only retrieved in the soil under inoculated trees at the Fresnes-sur-Escaut site. While no marked differences between sites and treatments were visible at the phylum level, analysis at the class level showed that the two soils had different fungal compositions. *Sordariomycetes, Leotiomycetes, Dothideomycetes, Eurotiomycetes* represented a lower proportion in the Fresnes-sur-Escaut soil than in the Pierrelaye soil, while *Agaricomycetes* and *Mortierellomycetes* were more abundant in the Fresnes-sur-Escaut soil (**Figure 3B**). Moreover, comparing the OTU dataset with the initial mycorrhizal species inoculated at the plantation stage, about half of these were detected after 7 years, namely, *Rhizophagus intradices*, *R. irregularis*, *Clarodeoglomus claroideum* and *C. etunicatum* (the AMF), and *Hebeloma mesophaeum* and *Laccaria proxima* (the ECM).

**Figure 4:**
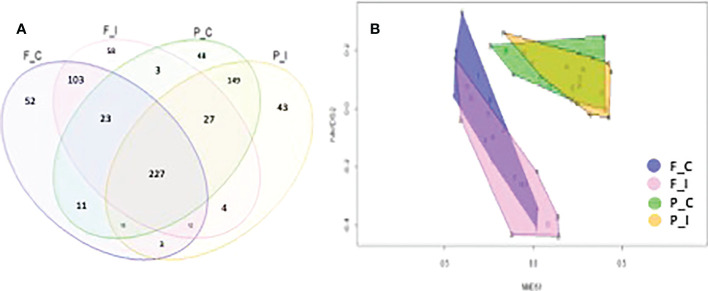
**(A)** Overlap of the different fungal communities from the two sites (Fresnes-sur-Escaut, F; Pierrelaye, P) and two treatments (control, C; inoculated, I) based on the OTUs (97% similarity). The number of OTUs shared by all the communities were 227. **(B)** NMDS plot of the fungal community structure for the two sites using the Bray-Curtis dissimilarity measure. Each point represents the fungal community of a given sample. Confidence area of the convex hull = 0.95.

To obtain a global view of the total fungal OTUs that were observed, the fungal communities of different sites and treatments are represented in a Venn diagram (**Figure 4A**). The sums of fungal OTUs observed in the four samples were 449, 457, 606 and 483 for the F_C, F_I, P_C and P_I treatments, respectively. The total number of OTUs in the present study was 781, while only 227 (29% of the total OTUs) were shared between the two sites (**Figure 4A**). As expected, the two treatments for a given soil, regardless of the site that was considered, shared the highest number of OTUs, especially for the Pierrelaye soil with a total of 421 OTUs (53%). OTUs that were specific to each community represented between 5 to 6% for the Pierrelaye site, and from 6 to 8% for the Fresnes-sur-Escaut site. More specifically, the highest proportion of unique OTUs was found in the inoculated treatment (F_I) for the Fresnes-sur-Escaut soil.

The importance of the habitat factor was corroborated by visual inspection of a two-dimension non-metric multidimensional scaling (NMDS) ordination; Bray-Curtis dissimilarity indicated that the fungal communities at the two sites were well separated (**Figure 4B**). Dispersion of the sample points was always lower at Pierrelaye than at Fresnes-sur-Escaut, demonstrating a greater homogeneity of the distribution of species at the Pierrelaye site (see size of the convex hulls in **Figure 4B**). Yet, the two treatments for the two sites overlapped substantially, demonstrating the high degree of similarity between the communities in the two different treatments.

**Figure 5:**
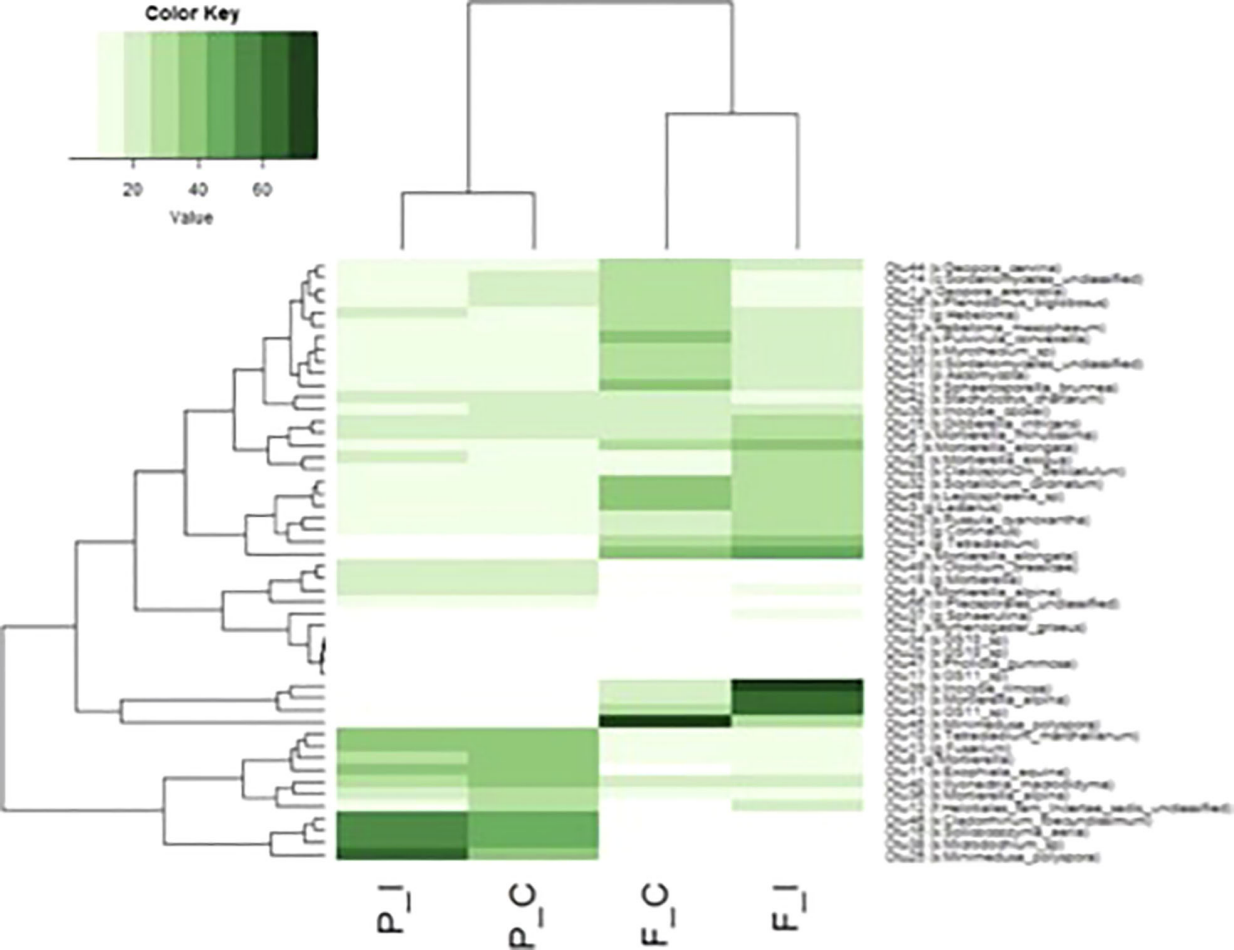
Comparison of the abundance of fungal OTUs among the 50 most abundant OTUs in at least one of the two sites (Fresnes-sur-Escaut, F; Pierrelaye, P) and two treatments (control, C; inoculated, I). The dendrogram represents linkage clustering using Euclidean distance measures. OTU delineation was based as the cutoff of 97% sequence similarity. Assignements between brackets indicate the lower taxonomic level associated with the OTU using the Greengenes database, k, kingdom; p, phylum; o, order; c, class; f, family; s, genus and species.

A heatmap was constructed with the 50 most abundant OTUs in each of the two treatments for the two sites and showed clear clustering between the fungal communities of the two sites and a strong homogeneity between the communities of the control and inoculated samples (**Figure 5**). Among these 50 OTUs, 11 were unique to the Pierrelaye site, while only 4 were unique to the Fresnes-sur-Escaut site, although they were not apportioned equally between the two treatments.

In terms of more specific details, four OTUs for the Pierrelaye site that were represented by OTU00025 (*Minimedusa polyspora)*, OTU00038 (*Microdochium* sp.), OUT00016 (*Solicoccozyma aeria*) and OTU00046 (*Cladorrhinum foecundissinum*) were highly abundant in inoculated samples. At the Fresnes-sur-Escaut site, OTU00045 (*Minimedusa polyspora*) was more abundant in control samples, while OTU00043 (*GS11*), OTU00031 (*Mortierella alpina*) and OTU00039 (*Inocybe rimosa*) were more abundant in the inoculated treatment.

### Analysis of the ectomycorrhizal and endophytic fungal communities

Each fungal OTU was assigned to a functional group using FUNguild (http://www.stbates.org/guilds/app.php) (Nguyen et al., 2015). For each assignment, the FUNguild tool provides a confidence ranking, while referring to previously peer-reviewed data (**Online Resource 3**). To examine the distribution of OTUs within the functional categories, the abundance of different groups of OTUs was set to 100% and OTUs were categorized into guilds (**Online Resource 4**). This analysis showed a dominance of OTUs that were assigned to the ECM fungus group for the Fresnes-sur-Escaut samples, reaching values between 45 and 33% for F_C and F_I, respectively, which accords with the observed high degree of mycorrhization (**Online Resource 4**). The second group of dominant OTUs was assigned to the endophyte group, with similar values between the two sites and treatments (around 20%), except for F_I, which reached 28%, followed by the undefined saprotroph group (10-13%) (**Online Resource 4**). The last most abundant group was “others”. It included all groups that were present with a percentage < 1% and was characterized mostly by AM fungi (**Online Resource 4**). Finally, endophyte-plant pathogens were observed only in the Pierrelaye soil for the two treatments, while animal pathogens were only found in the inoculated soil at Fresnes-sur-Escaut (**Online Resource 4**).

## Discussion

With respect to TE accumulation in perennial tissues, results of the present study agree with our earliest data (Phanthavongsa et al., 2017), in which any differences in TE content of Skado leaves between the two treatments were observed on moderately contaminated soils. Bissonnette et al. (2010) similarly showed that, on a slightly contaminated site, inoculation using AMF had no significant effect on Cd accumulation in poplars. Yet, the data that have been presented here reinforce the observation that the bark tissues had much higher element and TE concentrations compared with the wood. Wood chips from SRC can be used as recycling mulching materials to enhance soil fertility and, therefore, there is a concern regarding valorizing bark tissues from species in the Salicaceae being grown on TE-contaminated soils (Vandecasteele et al., 2013). However, Chalot et al. (2020) and Evangelou et al. (2012) demonstrated that harvesting poplar or *Salix* biomass, in which a smaller bark proportion compared to trunk, would be suitable.

Recent efforts have been made to characterize the microbiome of the plant rhizosphere using high throughput sequencing (Cregger et al., 2018; Durand et al., 2018; Alvarez-Lopez et al., 2020; Yung et al., 2021). In the present study, the fungal communities of a poplar plantation at the two study sites were characterized by metabarcoding tools using Illumina MiSeq sequencing platform. As previously stated, if we compare the OTU dataset with the initial mycorrhizal species that were introduced at the plantation stage, about half of the inoculated species were detected after 7 years: *Rhizophagus intradices*, *R. irregularis*, *Clarodeoglomus claroideum* and *C. etunicatum*, as AMF; and *Hebeloma mesophaeum* and *Laccaria proxima*, as ECM. Previous findings (Krpata et al., 2008; Regvar et al., 2010; Hrynkiewicz et al., 2012) demonstrated that these AMF and ECM strains are often associated with different poplar clones, especially under stressful conditions, such as TE contamination. For instance, *R. intradices* demonstrated high capacity to improve TE tolerance to tomato (*Solanum lycopersicum*), maize (*Zea mays*) or barrel medic (*Medicago truncatula*) (Hildebrandt et al., 2007) and to increase plant biomass (Ciccatelli et al., 2010) in highly stressful and nutrient-poor environments.

Rarefaction analyses and richness indices revealed that much of the total diversity detectable with Illumina-based sequencing was obtained. The demonstration of higher richness and diversity in the Pierrelaye site (a sandy site with lower concentrations of TE) compared to the Fresnes-sur-Escaut site (a silt-loam site with higher concentrations of TE) was consistent with the previous study of Foulon et al. (2016a), in which the Pierrelaye soil exhibited high richness and diversity indices.

All of our data revealed that the Pierrelaye site had a higher level of diversity that was associated with a higher percentage of OTUs, a large part of which was shared between the two treatments (53%) and only a small percentage, ranging from 5 and 6%, was unique for each treatment. The fungal community at the Fresnes-sur-Escaut site was characterized by fewer differences and lower homogeneity between the populations of the two treatments. Overall, these results indicate that soil characteristics appear to be the main drivers (rather than inoculation) affecting the structure of fungal communities. Indeed, the soil physicochemical parameters, including texture and calcium concentration, seem to be determining factors driving the composition of fungal communities and networks in poplar roots, as has been previously demonstrated (Bonito et al., 2014; Cregger et al., 2018; Bonito et al., 2019).

Johnson et al. (2013) observed that more favourable soil conditions, in terms of pH, OM, N and texture, as described for the Fresnes-sur-Escaut site with a lower diversity of mycorrhizal fungi, are explained by both optimal allocation of resource and biotic interactions. In addition, another study (Huang et al., 2019) have shown that plants living under favourable conditions do not require a high diversity of mycorrhizal species; in contrast, they compete strongly for nutrients and OM, potentially leading to a decrease in diversity. There is a broad consensus that excessive concentrations of heavy metals, such as Cd, Zn and Pb, have substantial negative effects on the abundance and diversity of fungi (Castañón-Silva et al., 2013; Yang et al., 2015; Taleski et al., 2020; Song et al., 2021). Indeed, the two soils exhibited high concentrations of Cd, Cu, Pb and Zn (Ciadamidaro et al., 2017) and a high degree of spatial heterogeneity in the concentration ranges of these metals. In general, total concentrations of TE were higher at Fresnes-sur-Escaut compared to Pierrelaye (see Ciadamidaro et al., 2017); this could have had negative effects on the richness and diversity of ECM communities.

Moreover, we established that fungal communities that were associated with the bulk soil under the plantation of the Skado cultivar at the two sites and treatments consisted mainly of ECM fungi and endophytes. ECM fungi exhibited a higher percentage of the compositions than did endophytes at the Fresnes-sur-Escaut site, but had a percentage that was identical to endophytes sampled at the Pierrelaye site. ECMs are known to create mutually beneficial interactions with their hosts, and to affect soil bioavailability and TE uptake by plants (Jentschke and Godbold, 2000). The fact that a majority of ECM were observed in our polluted soils could be due to the significant development of ECM fungi under stressful conditions, such as TE contamination. Indeed, previous study (Bellion et al., 2006) has shown a strong association of ECM fungi with different clones of *Populus* in the presence of high TE concentrations and demonstrated that ECM protect themselves and their hosts from TE pollution, for example, by binding them into cell-wall components or by sequestrating large amounts of TE in vacuoles. This symbiosis between ECM fungi and their hosts in TE polluted sites often leads to an increase in surface area that was potentially available for nutrient and water uptake (Rousseau et al., 1994). In our study, this hypothesis is greatly supported by an increase of biomass for the inoculated Skado poplars over time at the two sites.

Moreover, the presence of endophytes in degraded environments has been well discussed in the last decade. It is recognized that endophytic fungi generally have positive impacts on plant communities, increasing plant fitness by conferring tolerance to abiotic and biotic stresses (Hubbard et al., 2014; Lacercat-Didier et al., 2016), increasing biomass, plant growth and yield by increasing nutrient uptake or suppressing phytopathogens through antifungal activity (Waqas et al., 2015; Shahabivand et al., 2017; Lata et al., 2018). Such a positive influence of endophytic fungi on host plants is attributed to the production of phytobeneficial substances, such as ACC-deaminase, auxins, siderophores, and extracellular polymeric substances, and to the solubilization of soil nutrients, which promote active plant responses against different stresses (Khan et al., 2015; Deng and Cao, 2017; Bilal et al., 2018). In our study, the symbiotic association of poplar with these two important groups of fungi could have favoured marked plant development and tolerance of the Skado clone to TE contamination.

Finally, our results demonstrate that mycorrhizal inoculation did not drastically modify the soil mycobiomes, as exhibited by the lack of significant differences between the inoculated and non-inoculated treatments, whether this is measured in terms of abundance or structure of the fungal communities. This may be explained by the initial presence of these strains at the two sites. Indeed, previous experiments that were conducted at Pierrelaye and Fresnes-sur-Escaut before and after mycorrhizal fungal inoculation (Foulon et al., 2016a; Foulon et al., 2016b; Yung et al., 2021) already found a predominance of the phyla Ascomycota and Basidiomycota with different percentages, more precisely *Geopora* sp. and *Lactarius* sp. at gender level. Regarding the other phyla, our results did not accord with previous work (Foulon et al., 2016a; Foulon et al., 2016b; Yung et al., 2021), bringing us to conclude that fungal populations evolved over time and that mycorrhizal inoculation affected fungal structure in the two sites. Moreover, a previous study (Egidi et al., 2019) observed that the Ascomycota exhibited significant higher frequency of genomic traits associated with both stress-tolerance and competitive abilities, such as melanin deposition, as well as resistance to antibiotics and antibiotic production.

Finally, the study presented here improves upon and extends the previous dataset that was created by Ciadamidaro et al. (2017). Indeed, during our previous dendrometric measurements (2013 and 2015), we determined that the inoculation treatment significantly increased the biomass yield relative to un-inoculated controls (expressed as t/ha/year) of Skado for the Pierrelaye and Fresnes-sur-Escaut sites (Ciadamidaro et al., 2017). This study demonstrates that the inoculum introduced during planting still exerts a positive effect on the productivity of poplar plots, also after 7 growing seasons. This growth period corresponds to a short rotation coppice (SRC) scenario, which has ecologically advantageous effects on soil physical properties of the topsoil (Kahle and Janssen, 2020). The results obtained from this study proved that the biofertilization approach applied at two contrasting contaminated sites, which is based upon mycorrhizal consortia, can be recommended as a promising agrotechnology for poplar-based agroforestry, which is considered as an appropriate system already used for the production of biofuels in rural areas (Gruenewald et al., 2007; Berhongaray et al., 2017).

## Data availability statement

The data presented in the study are deposited in the ENA repository, accession number PRJEB56688.

## Author contributions

Conceptualization: MC, DK and DB. Methodology: LC, VB, MC and DB. Software: LC and SP. Validation: LC and MC. Formal analysis: LC, SP, PB, CZ, OG and VB. Investigation: LC and SP. Resources: MC. Data curation: LC and SP. Writing—original draft preparation: LC. Writing—review and editing: LC, PB, DK, DB and MC. Visualization: LC. Supervision: MC. Project administration: MC. Funding acquisition: MC and DK. All authors have read and agreed to the published version of the manuscript. All authors contributed to the article and approved the submitted version.

## Funding

This research was funded by Agence Nationale de la Recherche (ANR), grant number ANR-10-INTB-1703-01-BIOFILTREE” and Natural Sciences and Engineering Research Council of Canada (NSERC) Strategic Project Grant to DK, grant number STPGP 396879-2010. “The APC was funded by ANR.

## Acknowledgments

We thank Voies Navigables de France (VNF) for allowing access to the Fresnes site and WFJ PARSONS for the English revision.

## Conflict of interest

The authors declare that the research was conducted in the absence of any commercial or financial relationships that could be construed as a potential conflict of interest.

## Publisher’s note

All claims expressed in this article are solely those of the authors and do not necessarily represent those of their affiliated organizations, or those of the publisher, the editors and the reviewers. Any product that may be evaluated in this article, or claim that may be made by its manufacturer, is not guaranteed or endorsed by the publisher.
