# Supplementary material for: Mycorrhizal inoculation effects on growth and the mycobiome of poplar on two phytomanaged sites after 7-year-short rotation coppicing
Source: Front Plant Sci. 2022 Oct 28;13:993301. doi: 10.3389/fpls.2022.993301 (PMC9650387; doi:10.3389/fpls.2022.993301)
Supplement: Supplementary file 1 [file DataSheet_1.docx]

**Online Resource 1** Nutrient and Trace Element concentrations (μg g^1^ DW) in bark and debarked-trunk of trees grown on the two study sites (Fresnes-sur-Escaut and Pierrelaye) for the two treatments (control and inoculated).

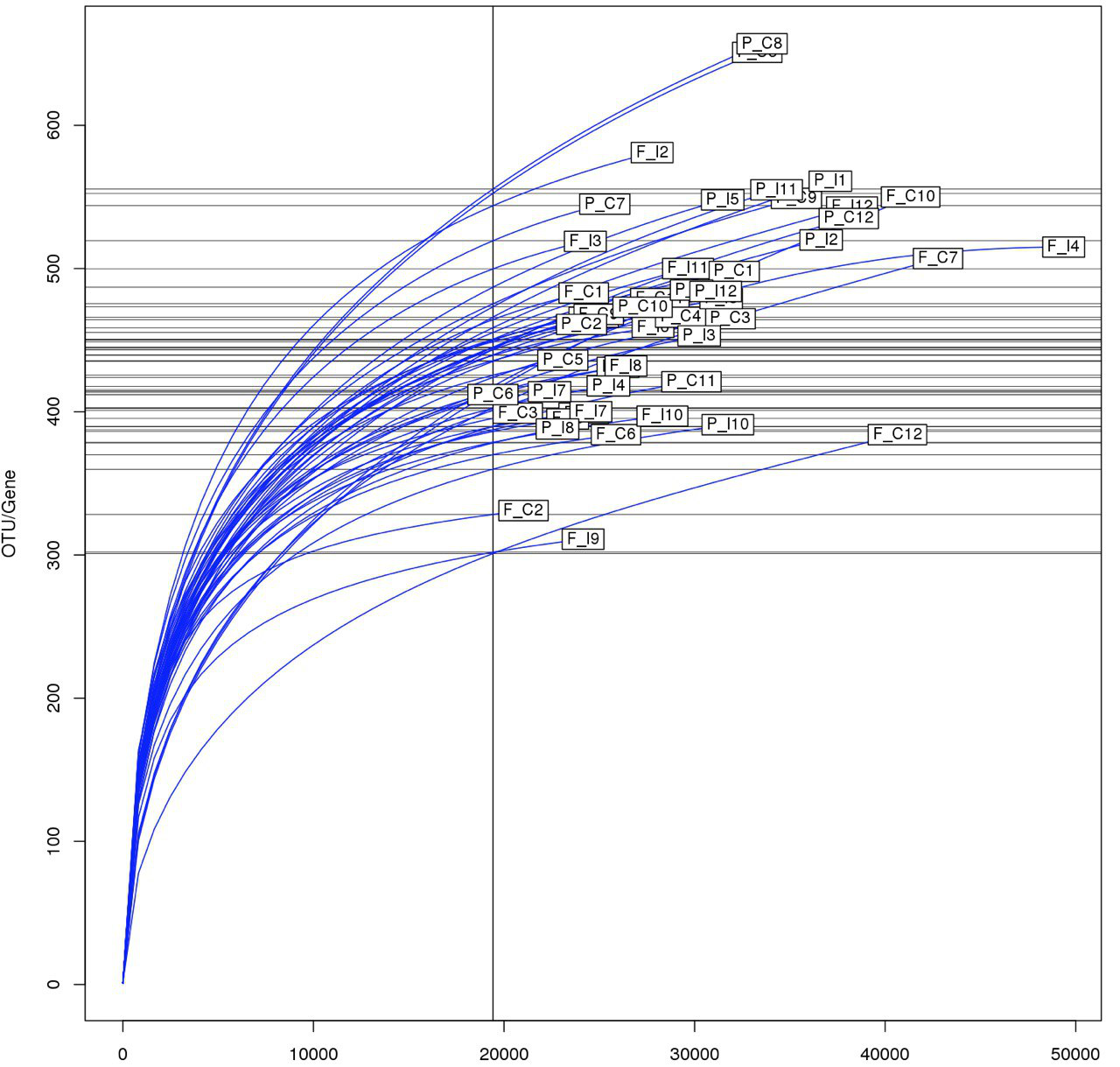


**Online Resource 2** Rarefaction analysis of ITS1 region sequence data for estimating fungal diversity based on a threshold of < 97% sequence similarity for the delineation of operational taxonomic units (OTUs). Comparisons of the fungal communities from the two study soils (Fresnes-sur-Escaut and Pierrelaye) and two treatments (control and inoculated) of *Populus* Skado field trials.

**Online Resource 3** Example of FUNguild assignment table

**Online Resource 4** Relative proportion of fungal sequences from the two sites (Fresnes-sur-Escaut: F; Pierrelaye: P) and the two treatments (control: C; inoculated: I) assigned to the major fungal guilds.
